# Supplementary material for: Inter- and intra-animal variation in the integrative properties of stellate cells in the medial entorhinal cortex
Source: eLife. 2020 Feb 13;9:e52258. doi: 10.7554/eLife.52258 (PMC7067584; doi:10.7554/eLife.52258)
Supplement: Supplementary file 1. — Analyses are as described for Table 1. Data are from GFP-positive putative pyramidal neurons (n = 42, N = 3). [file elife-52258-supp1.docx]

| **Property** | **Slope** | **p (slope)** | **Marginal R2** | **Conditional R2** | **Slope (min)** | **Slope (max)** | **p (vs linear)** |
| --- | --- | --- | --- | --- | --- | --- | --- |
| Vm | 2.0086 | 0.1558 | 0.1104 | 0.1104 | 2.0086 | 2.0086 | 1 |
| IR | 0.1655 | 0.9652 | 0.0001 | 0.0181 | 0.1655 | 0.1655 | 1 |
| Sag | -0.0137 | 0.5928 | 0.0142 | 0.0142 | -0.0137 | -0.0137 | 1 |
| Tm | 3.0716 | 0.2531 | 0.0861 | 0.1681 | 1.7960 | 4.2054 | 1 |
| Res. frequency | 0.2200 | 0.8135 | 0.0042 | 0.0042 | 0.2200 | 0.2200 | 1 |
| Res. magnitude | 0.0940 | 0.3552 | 0.0673 | 0.1764 | 0.0623 | 0.1553 | 1 |
| Spike thresold | 2.5595 | 0.1558 | 0.0986 | 0.0986 | 2.5595 | 2.5595 | 1 |
| Spike maximum | 1.1065 | 0.4628 | 0.0280 | 0.1533 | 1.1065 | 1.1065 | 1 |
| Spike width | 0.0495 | 0.0298 | 0.1922 | 0.1922 | 0.0495 | 0.0495 | 1 |
| Rheobase | -15.4864 | 0.5928 | 0.0165 | 0.0165 | -15.4864 | -15.4864 | 1 |
| Spike AHP | 1.6079 | 0.3742 | 0.0470 | 0.0982 | 1.0252 | 2.2523 | 1 |
| I-F slope | -0.0037 | 0.8625 | 0.0019 | 0.0019 | -0.0037 | -0.0037 | 1 |
