# Supplementary material for: Inter- and intra-animal variation in the integrative properties of stellate cells in the medial entorhinal cortex
Source: eLife. 2020 Feb 13;9:e52258. doi: 10.7554/eLife.52258 (PMC7067584; doi:10.7554/eLife.52258)
Supplement: Supplementary file 2. — The distinguishing electrophysiological features of SCs and their dorsoventral organization were apparent at all ages, with some features depending significantly on age (left columns), consistent with the idea that SCs continue to mature beyond P18 (Boehlen et al., 2010; Burton et al., 2008). When we considered only animals between P33 and P44, we did not find any significant effect of age (right columns). Significance estimates for the effects of dorsoventral position (dvloc), age (age) and interactions between dorsoventral position and age (dvloc:age) were estimated using type II ANOVA and Wald χ2 test from fits to mixed models containing age and location as fixed effects and animal identity as random effects. Significance estimates were adjusted for multiple comparisons using the Benjamini and Hochberg method. [file elife-52258-supp2.docx]

|  | **All ages** | | | | | **P32 < age** | | | | | **P32 < age < P45** | | | | |
| --- | --- | --- | --- | --- | --- | --- | --- | --- | --- | --- | --- | --- | --- | --- | --- |
| **property** | **dvloc** | **age** | **dvloc:age** | **N** | **n** | **dvloc** | **age** | **dvloc:age** | **N** | **n** | **dvloc** | **age** | **dvloc:age** | **N** | **n** |
| Vm (mV) | 3.32e-07 | 0.557568 | 0.0833 | 27 | 836 | 1.65e-07 | 0.869 | 0.00263 | 26 | 795 | 4.44e-06 | 0.691 | 0.732 | 25 | 779 |
| IR (MΩ) | 5.03e-75 | 0.000804 | 0.6173 | 27 | 836 | 3.54e-81 | 0.468 | 0.42608 | 26 | 795 | 2.86e-73 | 0.985 | 0.732 | 25 | 779 |
| Sag | 4.38e-19 | 0.004268 | 0.0833 | 27 | 836 | 1.06e-20 | 0.468 | 0.02551 | 26 | 795 | 7.85e-18 | 0.691 | 0.732 | 25 | 779 |
| Tm (ms) | 5.19e-28 | 0.091903 | 0.9044 | 27 | 836 | 1.45e-28 | 0.869 | 0.96718 | 26 | 795 | 4.16e-28 | 0.844 | 0.732 | 25 | 779 |
| Res. frequency (Hz) | 1.37e-17 | 0.006573 | 0.1811 | 27 | 836 | 7.11e-21 | 0.468 | 0.00242 | 26 | 795 | 1.54e-17 | 0.985 | 0.732 | 25 | 779 |
| Res. magnitude | 5.78e-12 | 0.006573 | 0.2673 | 27 | 836 | 4.42e-13 | 0.869 | 0.16209 | 26 | 795 | 7.83e-12 | 0.691 | 0.732 | 25 | 779 |
| Spike thresold (mV) | 8.39e-01 | 0.648355 | 0.6173 | 27 | 836 | 8.47e-01 | 0.869 | 0.42924 | 26 | 795 | 6.70e-01 | 0.985 | 0.806 | 25 | 779 |
| Spike maximum (mV) | 3.97e-08 | 0.784802 | 0.6678 | 27 | 836 | 1.96e-07 | 0.869 | 0.67151 | 26 | 795 | 3.98e-07 | 0.985 | 0.732 | 25 | 779 |
| Spike width (ms) | 1.31e-02 | 0.053866 | 0.9182 | 27 | 836 | 2.26e-02 | 0.869 | 0.43512 | 26 | 795 | 4.21e-02 | 0.985 | 0.732 | 25 | 779 |
| Rheobase (pA) | 4.15e-79 | 0.247452 | 0.6173 | 27 | 836 | 2.62e-73 | 0.869 | 0.96718 | 26 | 795 | 2.23e-71 | 0.691 | 0.732 | 25 | 779 |
| Spike AHP (mV) | 1.14e-02 | 0.648355 | 0.9182 | 27 | 836 | 1.67e-02 | 0.579 | 0.97669 | 26 | 795 | 2.31e-02 | 0.691 | 0.891 | 25 | 779 |
| I-F slope (Hz/pA) | 6.47e-21 | 0.257798 | 0.3015 | 27 | 692 | 5.64e-21 | 0.468 | 0.42924 | 26 | 666 | 7.25e-19 | 0.691 | 0.732 | 25 | 656 |
