# Supplementary material for: Inter- and intra-animal variation in the integrative properties of stellate cells in the medial entorhinal cortex
Source: eLife. 2020 Feb 13;9:e52258. doi: 10.7554/eLife.52258 (PMC7067584; doi:10.7554/eLife.52258)
Supplement: Supplementary file 3. — Analyses suggesting that the membrane potential sag, resonance frequency, and spike half-width of SCs differ between mice housed in standard and large home cages. Significance estimates for the effects of dorsoventral position (dvloc), housing (housing) and interactions between dorsoventral position and housing (dvloc:housing) estimated using type II ANOVA and Wald χ2 test from fits to mixed models containing age and location as fixed effects and animal identity as random effects. Initial significance estimates (raw p) were adjusted for multiple comparisons (adjusted p) using the Benjamini and Hochberg method. [file elife-52258-supp3.docx]

|  | | | **Fixed effects** | | | | **raw p** | | | **adjusted p** | | |
| --- | --- | --- | --- | --- | --- | --- | --- | --- | --- | --- | --- | --- |
| **property** | **N** | **n** | **Int** | **dvloc** | **housing** | **dv:housing** | **dvloc** | **housing** | **dv:housing** | **dvloc_adj** | **housing_adj** | **dv:housing_adj** |
| Vm (mV) | 25 | 779 | -63.657 | -0.836 | 0.276 | 0.0524 | 6.1e-06 | 0.5330 | 0.8872 | 8.2e-06 | 0.581 | 0.972 |
| IR (MΩ) | 25 | 779 | 20.015 | 11.288 | -2.306 | 0.3681 | 7.1e-74 | 0.0942 | 0.7806 | 4.3e-73 | 0.188 | 0.972 |
| Sag | 25 | 779 | 0.531 | 0.032 | 0.023 | 0.0009 | 3.0e-19 | 0.0031 | 0.9042 | 8.9e-19 | 0.031 | 0.972 |
| Tm (ms) | 25 | 779 | 8.408 | 2.301 | -1.110 | 0.4297 | 9.1e-28 | 0.0606 | 0.3824 | 3.7e-27 | 0.145 | 0.765 |
| Res. frequency (Hz) | 25 | 779 | 9.187 | -1.024 | 1.189 | -0.2756 | 3.9e-17 | 0.0113 | 0.3543 | 7.8e-17 | 0.045 | 0.765 |
| Res. magnitude | 25 | 779 | 1.804 | -0.091 | 0.048 | -0.0339 | 8.7e-12 | 0.7457 | 0.3254 | 1.5e-11 | 0.746 | 0.765 |
| Spike thresold (mV) | 25 | 779 | -39.222 | 0.140 | 0.715 | 0.0276 | 6.8e-01 | 0.4333 | 0.9723 | 6.8e-01 | 0.537 | 0.972 |
| Spike maximum (mV) | 25 | 779 | 43.696 | 2.343 | 1.073 | -0.6997 | 3.3e-07 | 0.4478 | 0.3664 | 5.0e-07 | 0.537 | 0.765 |
| Spike width (ms) | 25 | 779 | 0.501 | 0.022 | -0.055 | -0.0136 | 4.0e-02 | 0.0052 | 0.3060 | 4.4e-02 | 0.031 | 0.765 |
| Rheobase (pA) | 25 | 779 | 398.932 | -97.192 | 65.600 | -35.5380 | 7.2e-96 | 0.0275 | 0.0031 | 8.7e-95 | 0.083 | 0.038 |
| Spike AHP (mV) | 25 | 779 | -56.474 | -0.470 | 0.980 | -0.2298 | 2.1e-02 | 0.2537 | 0.6794 | 2.5e-02 | 0.380 | 0.972 |
| I-F slope (Hz/pA) | 25 | 656 | 0.034 | 0.039 | 0.011 | -0.0032 | 5.0e-19 | 0.1875 | 0.7108 | 1.2e-18 | 0.321 | 0.972 |
