# Supplementary material for: Inter- and intra-animal variation in the integrative properties of stellate cells in the medial entorhinal cortex
Source: eLife. 2020 Feb 13;9:e52258. doi: 10.7554/eLife.52258 (PMC7067584; doi:10.7554/eLife.52258)
Supplement: Supplementary file 4. — Results from comparison of a mixed effect model incorporating dorsoventral location and housing with an equivalent linear model. The significance estimate (p) is calculated using a χ2test and adjusted for multiple comparisons (p_adj) using the Benjamini and Hochberg method. [file elife-52258-supp4.docx]

| **property** | **deviance (mixed)** | **deviance (linear)** | **df (mixed)** | **df (linear)** | **p** | **p_adj** |
| --- | --- | --- | --- | --- | --- | --- |
| Vm (mV) | 3216.17 | 3368.84 | 7 | 5 | 7.05e-34 | 2.12e-33 |
| IR (MΩ) | 5212.59 | 5309.83 | 7 | 5 | 7.65e-22 | 1.31e-21 |
| Sag | -2740.77 | -2645.37 | 7 | 5 | 1.92e-21 | 2.88e-21 |
| Tm (ms) | 3704.02 | 3786.67 | 7 | 5 | 1.13e-18 | 1.50e-18 |
| Res. frequency (Hz) | 2801.35 | 2955.21 | 7 | 5 | 3.91e-34 | 1.56e-33 |
| Res. magnitude | -81.94 | -50.33 | 7 | 5 | 1.37e-07 | 1.37e-07 |
| Spike thresold (mV) | 3814.26 | 3861.51 | 7 | 5 | 5.48e-11 | 5.98e-11 |
| Spike maximum (mV) | 4071.15 | 4274.50 | 7 | 5 | 6.99e-45 | 4.19e-44 |
| Spike width (ms) | -2237.45 | -1943.78 | 7 | 5 | 1.70e-64 | 2.04e-63 |
| Rheobase (pA) | 8742.68 | 8885.93 | 7 | 5 | 7.83e-32 | 1.57e-31 |
| Spike AHP (mV) | 3909.56 | 3977.71 | 7 | 5 | 1.59e-15 | 1.91e-15 |
| I-F slope (Hz/pA) | -2766.12 | -2622.51 | 7 | 5 | 6.55e-32 | 1.57e-31 |
