# Supplementary material for: Inter- and intra-animal variation in the integrative properties of stellate cells in the medial entorhinal cortex
Source: eLife. 2020 Feb 13;9:e52258. doi: 10.7554/eLife.52258 (PMC7067584; doi:10.7554/eLife.52258)
Supplement: Supplementary file 6. — Mediolateral as well as dorsoventral position has been reported to determine the sub-threshold electrophysiological features of SCs (Canto and Witter, 2012). We found significant effects of mediolateral position on all measured electrophysiological features. However, the sizes of the effects of mediolateral position on subthreshold features (vm, ir, sag, tau, resf, resmag, and rheo) were much smaller than for dorsoventral position. By contrast, supra-threshold features (spkthr, spkmax, and ahp) were more greatly affected by mediolateral position, with more medial neurons having a higher spike threshold, and lower amplitudes of the spike peak and of after-hyperpolarization. Fixed effects are the intercept and slope coefficients for mixed models containing dorsoventral and mediolateral location as fixed effects and animal identity as random effects. Significance estimates for the effects of dorsoventral position (dvloc), mediolateral position (ml) and interactions between dorsoventral position and mediolateral position (dvloc:ml) are estimated using type II ANOVA and Wald χ2 tests from the fits of the mixed models. Initial significance estimates (raw p) were adjusted for multiple comparisons (adjusted p) using the Benjamini and Hochberg method. [file elife-52258-supp6.docx]

|  | | | **Fixed effects** | | | | **raw p** | | | **adjusted p** | | |
| --- | --- | --- | --- | --- | --- | --- | --- | --- | --- | --- | --- | --- |
| **property** | **N** | **n** | **Int** | **dvloc** | **ml** | **dv:ml** | **dvloc** | **ml** | **dv:ml** | **dvloc_adj** | **ml_adj** | **dv:ml_adj** |
| Vm (mV) | 18 | 650 | -63.390 | -1.069 | -0.4528 | 0.8628 | 1.1e-03 | 4.9e-03 | 0.00531 | 1.5e-03 | 5.3e-03 | 0.0159 |
| IR (MΩ) | 18 | 650 | 16.951 | 12.533 | 3.0417 | -1.1816 | 1.6e-63 | 1.9e-03 | 0.28946 | 1.9e-62 | 2.9e-03 | 0.3859 |
| Sag | 18 | 650 | 0.547 | 0.036 | -0.0052 | -0.0092 | 1.2e-14 | 3.1e-05 | 0.16977 | 3.7e-14 | 5.3e-05 | 0.2547 |
| Tm (ms) | 18 | 650 | 7.659 | 3.027 | 0.8499 | -1.4678 | 5.6e-16 | 2.4e-03 | 0.00033 | 2.2e-15 | 3.2e-03 | 0.0020 |
| Res. frequency (Hz) | 18 | 650 | 9.552 | -1.307 | -0.0139 | 0.3652 | 1.0e-12 | 3.1e-03 | 0.12310 | 2.1e-12 | 3.7e-03 | 0.2110 |
| Res. magnitude | 18 | 650 | 1.847 | -0.154 | -0.0318 | 0.1077 | 1.8e-09 | 1.0e-05 | 0.00138 | 2.6e-09 | 2.0e-05 | 0.0055 |
| Spike thresold (mV) | 18 | 650 | -40.060 | 0.300 | 2.2350 | -0.1210 | 5.9e-01 | 1.2e-21 | 0.78973 | 5.9e-01 | 3.6e-21 | 0.8482 |
| Spike maximum (mV) | 18 | 650 | 46.435 | 2.376 | -3.6126 | -1.1149 | 5.4e-10 | 1.0e-107 | 0.00910 | 9.2e-10 | 1.2e-106 | 0.0218 |
| Spike width (ms) | 18 | 650 | 0.497 | 0.012 | -0.0107 | 0.0019 | 9.0e-02 | 8.6e-02 | 0.84817 | 9.8e-02 | 8.6e-02 | 0.8482 |
| Rheobase (pA) | 18 | 650 | 456.555 | -121.470 | -28.2342 | 3.1118 | 6.1e-35 | 4.7e-06 | 0.77997 | 3.6e-34 | 1.1e-05 | 0.8482 |
| Spike AHP (mV) | 18 | 650 | -56.987 | -1.126 | 1.8540 | 1.5587 | 8.0e-02 | 3.2e-57 | 0.00017 | 9.5e-02 | 1.9e-56 | 0.0020 |
| I-F slope (Hz/pA) | 18 | 548 | 0.027 | 0.033 | 0.0176 | 0.0118 | 2.6e-13 | 1.9e-33 | 0.01813 | 6.2e-13 | 7.4e-33 | 0.0363 |
