# Supplementary material for: Inter- and intra-animal variation in the integrative properties of stellate cells in the medial entorhinal cortex
Source: eLife. 2020 Feb 13;9:e52258. doi: 10.7554/eLife.52258 (PMC7067584; doi:10.7554/eLife.52258)
Supplement: Supplementary file 8. — We anticipated that the interval between slice preparation and recording may influence the measured electrophysiological features. Consistent with our expectation, analyses of the data were consistent with changes to some electrophysiological features of SCs with time since slice preparation, but dorsoventral gradients could not be explained by these changes. Significance estimates for the effects of dorsoventral position (dvloc), time since slice preparation (rect) and interactions between dorsoventral position and experimenter (dvloc:rect) estimated using type II ANOVA and Wald χ2 tests from fits to mixed models containing age and location as fixed effects and animal identity as random effects. Initial significance estimates (raw p) were adjusted for multiple comparisons (adjusted p) using the Benjamini and Hochberg method. [file elife-52258-supp8.docx]

|  | | | **Fixed effects** | | | | **raw p** | | | **adjusted p** | | |
| --- | --- | --- | --- | --- | --- | --- | --- | --- | --- | --- | --- | --- |
| **property** | **N** | **n** | **Int** | **dvloc** | **rect** | **dv:rect** | **dvloc** | **rect** | **dv:rect** | **dvloc_adj** | **rect_adj** | **dv:rect_adj** |
| Vm (mV) | 25 | 778 | -70.3538 | 0.425 | 1.0e+01 | -1.972 | 5.0e-07 | 2.2e-19 | 1.7e-01 | 6.8e-07 | 1.3e-18 | 4.0e-01 |
| IR (MΩ) | 25 | 778 | 14.8127 | 15.191 | 5.4e+00 | -5.535 | 5.6e-72 | 8.5e-01 | 3.0e-01 | 6.7e-71 | 9.3e-01 | 5.0e-01 |
| Sag | 25 | 778 | 0.5482 | 0.023 | -9.3e-04 | 0.013 | 1.2e-18 | 4.9e-01 | 6.8e-01 | 2.8e-18 | 5.9e-01 | 6.8e-01 |
| Tm (ms) | 25 | 778 | 8.2724 | 3.357 | -9.7e-01 | -1.142 | 2.7e-27 | 8.4e-02 | 5.8e-01 | 1.1e-26 | 1.3e-01 | 6.8e-01 |
| Res. frequency (Hz) | 25 | 778 | 7.2370 | -0.871 | 4.2e+00 | -0.569 | 2.8e-16 | 1.5e-06 | 6.2e-01 | 5.7e-16 | 3.6e-06 | 6.8e-01 |
| Res. magnitude | 25 | 778 | 1.5902 | -0.005 | 3.7e-01 | -0.167 | 7.4e-12 | 9.0e-02 | 3.4e-01 | 1.3e-11 | 1.3e-01 | 5.0e-01 |
| Spike thresold (mV) | 25 | 778 | -55.9008 | 6.033 | 2.6e+01 | -9.539 | 2.7e-01 | 5.8e-36 | 1.3e-06 | 2.7e-01 | 7.0e-35 | 1.5e-05 |
| Spike maximum (mV) | 25 | 778 | 40.7004 | -0.699 | 5.6e+00 | 3.866 | 5.1e-07 | 3.5e-09 | 1.3e-01 | 6.8e-07 | 1.0e-08 | 3.9e-01 |
| Spike width (ms) | 25 | 778 | 0.5921 | 0.045 | -2.0e-01 | -0.045 | 2.4e-02 | 4.9e-17 | 3.1e-01 | 2.6e-02 | 1.9e-16 | 5.0e-01 |
| Rheobase (pA) | 25 | 778 | 451.9604 | -98.908 | -1.3e+01 | -30.567 | 1.3e-66 | 1.7e-01 | 5.6e-01 | 7.6e-66 | 2.3e-01 | 6.8e-01 |
| Spike AHP (mV) | 25 | 778 | -60.7015 | 3.712 | 7.5e+00 | -6.633 | 2.0e-02 | 9.7e-01 | 4.3e-03 | 2.4e-02 | 9.7e-01 | 1.7e-02 |
| I-F slope (Hz/pA) | 25 | 655 | 0.0092 | 0.113 | 5.0e-02 | -0.115 | 1.1e-22 | 4.5e-06 | 2.6e-06 | 3.4e-22 | 9.1e-06 | 1.6e-05 |
