# Supplementary material for: Inter- and intra-animal variation in the integrative properties of stellate cells in the medial entorhinal cortex
Source: eLife. 2020 Feb 13;9:e52258. doi: 10.7554/eLife.52258 (PMC7067584; doi:10.7554/eLife.52258)
Supplement: Supplementary file 9. — In anticipation of the effects of the time since slice preparation on the electrophysiological features of SCS, we varied the direction along the dorsoventral axis from which consecutive recordings were made between experimenters and experimental days (see 'Materials and methods'). Consistent with effects of time on electrophysiological features (see Supplementary file 7 above), we found that the direction in which sequential recordings were made influenced the slope, but not the intercept of several electrophysiological features. Significance estimates for the effects of dorsoventral position (dvloc), direction in which sequential recordings were made (dir) and interactions between dorsoventral position and recording direction (dvloc:dir) estimated using type II ANOVA and Wald χ2 tests from fits to mixed models containing age and location as fixed effects and animal identity as random effects. Initial significance estimates (raw p) were adjusted for multiple comparisons (adjusted p) using the Benjamini and Hochberg method. [file elife-52258-supp9.docx]

|  | | | **Fixed effects** | | | | **raw p** | | | **adjusted p** | | |
| --- | --- | --- | --- | --- | --- | --- | --- | --- | --- | --- | --- | --- |
| **property** | **N** | **n** | **Int** | **dvloc** | **dir** | **dv:dir** | **dvloc** | **dir** | **dv:dir** | **dvloc_adj** | **dir_adj** | **dv:dir_adj** |
| Vm (mV) | 18 | 650 | -64.127 | -0.063 | 1.351 | -1.3535 | 2.8e-04 | 0.509 | 2.4e-05 | 3.7e-04 | 0.61 | 9.4e-05 |
| IR (MΩ) | 18 | 650 | 18.337 | 11.758 | 0.427 | 0.1689 | 8.7e-64 | 0.374 | 8.8e-01 | 1.0e-62 | 0.57 | 9.2e-01 |
| Sag | 18 | 650 | 0.534 | 0.040 | 0.021 | -0.0164 | 1.3e-16 | 0.411 | 1.9e-02 | 3.1e-16 | 0.57 | 3.8e-02 |
| Tm (ms) | 18 | 650 | 7.975 | 2.269 | 0.091 | 0.2595 | 2.5e-20 | 0.144 | 5.4e-01 | 1.0e-19 | 0.49 | 7.2e-01 |
| Res. frequency (Hz) | 18 | 650 | 9.372 | -0.905 | 0.443 | -0.5214 | 1.9e-12 | 0.429 | 3.6e-02 | 3.8e-12 | 0.57 | 6.3e-02 |
| Res. magnitude | 18 | 650 | 1.846 | -0.104 | -0.030 | -0.0035 | 1.0e-08 | 0.122 | 9.2e-01 | 1.7e-08 | 0.49 | 9.2e-01 |
| Spike thresold (mV) | 18 | 650 | -40.893 | 1.947 | 4.997 | -4.2461 | 5.2e-01 | 0.219 | 1.0e-23 | 5.2e-01 | 0.49 | 1.2e-22 |
| Spike maximum (mV) | 18 | 650 | 43.530 | 3.044 | 2.133 | -1.7908 | 5.0e-08 | 0.556 | 2.4e-03 | 7.4e-08 | 0.61 | 5.9e-03 |
| Spike width (ms) | 18 | 650 | 0.511 | -0.012 | -0.042 | 0.0491 | 1.2e-01 | 0.059 | 2.8e-06 | 1.3e-01 | 0.49 | 1.7e-05 |
| Rheobase (pA) | 18 | 650 | 439.068 | -119.614 | 10.707 | -1.5591 | 2.0e-43 | 0.168 | 9.0e-01 | 1.2e-42 | 0.49 | 9.2e-01 |
| Spike AHP (mV) | 18 | 650 | -56.018 | -0.474 | 0.408 | -0.4158 | 1.1e-02 | 0.872 | 4.0e-01 | 1.3e-02 | 0.87 | 6.0e-01 |
| I-F slope (Hz/pA) | 18 | 548 | 0.047 | 0.028 | -0.023 | 0.0189 | 7.5e-18 | 0.245 | 6.5e-04 | 2.2e-17 | 0.49 | 1.9e-03 |
