# Supplementary material for: Inter- and intra-animal variation in the integrative properties of stellate cells in the medial entorhinal cortex
Source: eLife. 2020 Feb 13;9:e52258. doi: 10.7554/eLife.52258 (PMC7067584; doi:10.7554/eLife.52258)
Supplement: Supplementary file 10. — Results from comparison of a mixed effect model incorporating dorsoventral location, housing, mediolateral position, experimenter identity and direction in which recordings were obtained with an equivalent linear model. Data are from animals between 32 and 45 days old. The significance estimate (p) is calculated using a χ2 test and adjusted for multiple comparisons (p_adj) using the Benjamini and Hochberg method. [file elife-52258-supp10.docx]

| **property** | **deviance (mixed)** | **deviance (linear)** | **df (mixed)** | **df (linear)** | **p** | **p_adj** |
| --- | --- | --- | --- | --- | --- | --- |
| Vm | 2636.81 | 2789.34 | 13 | 11 | 7.56e-34 | 3.02e-33 |
| IR | 4315.73 | 4407.44 | 13 | 11 | 1.22e-20 | 1.62e-20 |
| Sag | -2293.74 | -2212.48 | 13 | 11 | 2.27e-18 | 2.72e-18 |
| Tm | 2997.16 | 3122.25 | 13 | 11 | 6.86e-28 | 1.37e-27 |
| Res. frequency | 2122.53 | 2266.61 | 13 | 11 | 5.16e-32 | 1.55e-31 |
| Res. magnitude | -115.70 | -80.54 | 13 | 11 | 2.31e-08 | 2.31e-08 |
| Spike thresold | 2936.17 | 2974.72 | 13 | 11 | 4.25e-09 | 4.63e-09 |
| Spike maximum | 3008.01 | 3239.16 | 13 | 11 | 6.40e-51 | 7.68e-50 |
| Spike width | -2067.20 | -1911.08 | 13 | 11 | 1.26e-34 | 7.55e-34 |
| Rheobase | 7247.31 | 7388.78 | 13 | 11 | 1.91e-31 | 4.58e-31 |
| Spike AHP | 2832.78 | 2943.86 | 13 | 11 | 7.58e-25 | 1.14e-24 |
| I-F slope | -2473.53 | -2349.35 | 13 | 11 | 1.08e-27 | 1.85e-27 |
