# Supplementary material for: Inter- and intra-animal variation in the integrative properties of stellate cells in the medial entorhinal cortex
Source: eLife. 2020 Feb 13;9:e52258. doi: 10.7554/eLife.52258 (PMC7067584; doi:10.7554/eLife.52258)
Supplement: Supplementary file 11. — Results from comparison of mixed effect models with dorsoventral location as a fixed effect and animal identity as a random effect using minimal datasets obtained by either HP (upper) or DG (lower). Data are from animals between 32 and 45 days old. Because of the smaller size of these datasets, the statistical power to detect inter-animal variation is reduced. Nevertheless, in these analyses, the conditional R2 of the mixed model fit was again substantially higher than the marginal R2, and most (9/12) features were better fit by a mixed model compared to a corresponding linear model in both datasets. [file elife-52258-supp11.docx]

| **Property** | **Nobs** | **Ngrps** | **marginal.R2** | **Conditional.R2** | **Dev(mixed)** | **Dev(linear)** | **Dev_DF (mixed)** | **Dev_DF (linear)** | **p** | **p(adj)** |
| --- | --- | --- | --- | --- | --- | --- | --- | --- | --- | --- |
| Vm (mV) | 155 | 5 | 0.0206213 | 0.2653003 | 618 | 639 | 5 | 3 | 3.54e-05 | 6.07e-05 |
| IR (MΩ) | 155 | 5 | 0.4338606 | 0.5813283 | 962 | 991 | 5 | 3 | 6.41e-07 | 1.54e-06 |
| Sag | 155 | 5 | 0.1800554 | 0.4129771 | -576 | -543 | 5 | 3 | 5.57e-08 | 1.67e-07 |
| Tm (ms) | 155 | 5 | 0.0931844 | 0.0931844 | 696 | 696 | 5 | 3 | 1.00e+00 | 1.00e+00 |
| Res. frequency (Hz) | 155 | 5 | 0.0564421 | 0.2271175 | 531 | 551 | 5 | 3 | 6.28e-05 | 9.41e-05 |
| Res. magnitude | 155 | 5 | 0.0904403 | 0.2491295 | -39.5 | -24.1 | 5 | 3 | 4.59e-04 | 5.01e-04 |
| Spike thresold (mV) | 155 | 5 | 0.2037783 | 0.3875763 | 667 | 695 | 5 | 3 | 1.12e-06 | 2.24e-06 |
| Spike maximum (mV) | 155 | 5 | 0.2940305 | 0.4130672 | 639 | 655 | 5 | 3 | 4.12e-04 | 4.94e-04 |
| Spike width (ms) | 155 | 5 | 0.0298582 | 0.5313267 | -546 | -458 | 5 | 3 | 7.54e-20 | 9.05e-19 |
| Rheobase (pA) | 155 | 5 | 0.2126002 | 0.7326461 | 1736 | 1788 | 5 | 3 | 7.33e-12 | 4.40e-11 |
| Spike AHP (mV) | 155 | 5 | 0.0051775 | 0.1661239 | 549 | 566 | 5 | 3 | 2.12e-04 | 2.82e-04 |
| I-F slope (Hz/pA) | 138 | 5 | 0.3033252 | 0.6856220 | -778 | -741 | 5 | 3 | 9.29e-09 | 3.72e-08 |
| **Property** | **Nobs** | **Ngrps** | **marginal.R2** | **Conditional.R2** | **Dev(mixed)** | **Dev(linear)** | **Dev_DF (mixed)** | **Dev_DF (linear)** | **p** | **p(adj)** |
| Vm (mV) | 70 | 6 | 0.0938959 | 0.4186968 | 314 | 322 | 5 | 3 | 2.05e-02 | 2.73e-02 |
| IR (MΩ) | 70 | 6 | 0.1473580 | 0.5692115 | 475 | 502 | 5 | 3 | 2.05e-06 | 5.41e-06 |
| Sag | 70 | 6 | 0.0086794 | 0.2642029 | -223 | -212 | 5 | 3 | 5.73e-03 | 9.83e-03 |
| Tm (ms) | 70 | 6 | 0.0068137 | 0.0507559 | 327 | 327 | 5 | 3 | 7.95e-01 | 8.67e-01 |
| Res. frequency (Hz) | 70 | 6 | 0.0008116 | 0.4514622 | 203 | 229 | 5 | 3 | 2.25e-06 | 5.41e-06 |
| Res. magnitude | 70 | 6 | 0.0238201 | 0.3629621 | 14.5 | 28.8 | 5 | 3 | 7.62e-04 | 1.52e-03 |
| Spike thresold (mV) | 70 | 6 | 0.1837547 | 0.4722489 | 342 | 352 | 5 | 3 | 8.09e-03 | 1.21e-02 |
| Spike maximum (mV) | 70 | 6 | 0.0519267 | 0.0745947 | 356 | 356 | 5 | 3 | 9.62e-01 | 9.62e-01 |
| Spike width (ms) | 70 | 6 | 0.1474713 | 0.7119071 | -268 | -230 | 5 | 3 | 6.49e-09 | 3.89e-08 |
| Rheobase (pA) | 70 | 6 | 0.3428390 | 0.8415186 | 787 | 834 | 5 | 3 | 7.09e-11 | 8.51e-10 |
| Spike AHP (mV) | 70 | 6 | 0.0207732 | 0.1760733 | 332 | 336 | 5 | 3 | 1.35e-01 | 1.62e-01 |
| I-F slope (Hz/pA) | 60 | 6 | 0.2925095 | 0.7238371 | -285 | -248 | 5 | 3 | 1.00e-08 | 4.01e-08 |
