# Supplementary material for: Inter- and intra-animal variation in the integrative properties of stellate cells in the medial entorhinal cortex
Source: eLife. 2020 Feb 13;9:e52258. doi: 10.7554/eLife.52258 (PMC7067584; doi:10.7554/eLife.52258)
Supplement: Supplementary file 12. — Significance estimates for the effects of dorsoventral position (dvloc), number of recorded neurons (counts) and interactions between dorsoventral position and number of recorded neurons (dvloc:counts) estimated using type II ANOVA and Wald χ2 tests from fits to mixed models containing age and location as fixed effects and animal identity as random effects. Initial significance estimates (raw p) were adjusted for multiple comparisons (adjusted p) using the Benjamini and Hochberg method. [file elife-52258-supp12.docx]

|  | | | **Fixed effects** | | | | **raw p** | | | **adjusted p** | | |
| --- | --- | --- | --- | --- | --- | --- | --- | --- | --- | --- | --- | --- |
| **property** | **N** | **n** | **Int** | **dvloc** | **counts** | **dv:counts** | **dvloc** | **counts** | **dv:counts** | **dvloc_adj** | **counts_adj** | **dv:counts_adj** |
| Vm (mV) | 25 | 779 | -63.262 | -1.334 | -0.00470 | 1.5e-02 | 7.2e-06 | 0.72310 | 0.384 | 9.7e-06 | 0.9641 | 0.58 |
| IR (MΩ) | 25 | 779 | 20.165 | 8.241 | -0.04767 | 9.6e-02 | 1.1e-75 | 0.61555 | 0.113 | 1.4e-74 | 0.9233 | 0.23 |
| Sag | 25 | 779 | 0.568 | 0.010 | -0.00055 | 6.2e-04 | 8.3e-20 | 0.98893 | 0.080 | 2.5e-19 | 0.9889 | 0.23 |
| Tm (ms) | 25 | 779 | 5.964 | 3.987 | 0.04659 | -3.9e-02 | 8.6e-28 | 0.34148 | 0.092 | 3.4e-27 | 0.9233 | 0.23 |
| Res. frequency (Hz) | 25 | 779 | 12.067 | -1.880 | -0.06141 | 1.8e-02 | 1.2e-18 | 0.00231 | 0.194 | 2.8e-18 | 0.0139 | 0.33 |
| Res. magnitude | 25 | 779 | 1.777 | -0.066 | 0.00166 | -1.3e-03 | 5.3e-12 | 0.83262 | 0.433 | 9.1e-12 | 0.9889 | 0.58 |
| Spike thresold (mV) | 25 | 779 | -38.810 | 0.278 | 0.00217 | -3.4e-03 | 6.7e-01 | 0.98669 | 0.925 | 6.7e-01 | 0.9889 | 0.97 |
| Spike maximum (mV) | 25 | 779 | 44.731 | 0.051 | -0.00543 | 5.4e-02 | 1.5e-07 | 0.46923 | 0.117 | 2.2e-07 | 0.9233 | 0.23 |
| Spike width (ms) | 25 | 779 | 0.327 | 0.064 | 0.00408 | -1.4e-03 | 1.4e-02 | 0.00022 | 0.019 | 1.6e-02 | 0.0027 | 0.22 |
| Rheobase (pA) | 25 | 779 | 388.533 | -73.882 | 1.56591 | -1.3e+00 | 8.3e-74 | 0.45670 | 0.043 | 5.0e-73 | 0.9233 | 0.23 |
| Spike AHP (mV) | 25 | 779 | -56.673 | -0.185 | 0.02540 | -1.2e-02 | 2.7e-02 | 0.59589 | 0.659 | 2.9e-02 | 0.9233 | 0.79 |
| I-F slope (Hz/pA) | 25 | 656 | 0.052 | 0.036 | -0.00032 | 1.3e-05 | 5.5e-18 | 0.32094 | 0.973 | 1.1e-17 | 0.9233 | 0.97 |
