# Supplementary material for: Inter- and intra-animal variation in the integrative properties of stellate cells in the medial entorhinal cortex
Source: eLife. 2020 Feb 13;9:e52258. doi: 10.7554/eLife.52258 (PMC7067584; doi:10.7554/eLife.52258)
Supplement: Supplementary file 13. — Analyses of inter-animal differences focusing only on data from animals for which > 35 recordings were made (N = 11, n = 459). Comparison of marginal and conditional R2 values continued to indicate substantial inter-animal variance, and fits obtained with mixed models remained significantly different to fits that did not account for animal identity (p<4.4×10^−5^). Analyses are as for Supplementary file 1, but are restricted to experiments in which > 35 neurons were recorded from. [file elife-52258-supp13.docx]

| **property** | **ngrps** | **nobs** | **marginal.R2** | **conditional.R2** | **mm_vslinear_pdiff_adj** |
| --- | --- | --- | --- | --- | --- |
| Vm | 11 | 459 | 0.0568 | 0.373 | 2.77e-29 |
| IR | 11 | 459 | 0.5551 | 0.634 | 5.67e-13 |
| Sag | 11 | 459 | 0.1629 | 0.345 | 1.48e-13 |
| Tm | 11 | 459 | 0.2357 | 0.405 | 1.99e-16 |
| Res. frequency | 11 | 459 | 0.2809 | 0.496 | 2.42e-23 |
| Res. magnitude | 11 | 459 | 0.1392 | 0.239 | 1.08e-07 |
| Spike thresold | 11 | 459 | 0.3139 | 0.416 | 4.43e-05 |
| Spike maximum | 11 | 459 | 0.4810 | 0.703 | 4.63e-31 |
| Spike width | 11 | 459 | 0.3246 | 0.537 | 1.29e-20 |
| Rheobase | 11 | 459 | 0.5444 | 0.681 | 4.45e-26 |
| Spike AHP | 11 | 459 | 0.4594 | 0.568 | 7.63e-16 |
| I-F slope | 11 | 390 | 0.5159 | 0.742 | 1.33e-12 |
