# Supplementary material for: Inter- and intra-animal variation in the integrative properties of stellate cells in the medial entorhinal cortex
Source: eLife. 2020 Feb 13;9:e52258. doi: 10.7554/eLife.52258 (PMC7067584; doi:10.7554/eLife.52258)
Supplement: Supplementary file 14. — Analyses are as described for Table 1, but were applied to principal components of the electrophysiological features of SCs. [file elife-52258-supp14.docx]

| **Feature** | **Slope** | **p (slope)** | **Marginal R2** | **Conditional R2** | **Slope (min)** | **Slope (max)** | **p (vs linear)** |
| --- | --- | --- | --- | --- | --- | --- | --- |
| PC1 | -2.43039 | 1.09e-15 | 0.49546 | 0.73250 | -3.16508 | -2.07375 | 3.85e-37 |
| PC2 | 0.95274 | 1.05e-04 | 0.09300 | 0.80751 | -1.07715 | 2.11870 | 2.20e-97 |
| PC3 | 0.25634 | 1.73e-01 | 0.01187 | 0.51214 | -0.43529 | 1.11058 | 2.96e-38 |
| PC4 | -0.26629 | 2.53e-01 | 0.00941 | 0.79049 | -1.47231 | 1.06858 | 4.44e-63 |
| PC5 | -0.11523 | 4.01e-01 | 0.00390 | 0.43605 | -0.49011 | 0.44713 | 3.11e-24 |
| PC6 | -0.01593 | 9.03e-01 | 0.00006 | 0.64068 | -1.12576 | 1.08530 | 7.97e-31 |
| PC7 | -0.06543 | 4.90e-01 | 0.00249 | 0.05729 | -0.18346 | 0.07602 | 2.94e-02 |
| PC8 | -0.01011 | 9.03e-01 | 0.00007 | 0.23752 | -0.31346 | 0.24928 | 3.49e-10 |
| PC9 | -0.13548 | 3.95e-02 | 0.01617 | 0.18557 | -0.22543 | 0.08319 | 4.98e-12 |
| PC10 | -0.01408 | 9.03e-01 | 0.00031 | 0.04810 | -0.02747 | -0.00033 | 8.19e-02 |
| PC11 | -0.00545 | 9.03e-01 | 0.00007 | 0.02613 | -0.00794 | -0.00382 | 2.74e-01 |
| PC12 | -0.07795 | 1.50e-02 | 0.02402 | 0.08892 | -0.19333 | 0.01590 | 7.02e-04 |
