# Supplementary material for: Inter- and intra-animal variation in the integrative properties of stellate cells in the medial entorhinal cortex
Source: eLife. 2020 Feb 13;9:e52258. doi: 10.7554/eLife.52258 (PMC7067584; doi:10.7554/eLife.52258)
Supplement: Supplementary file 15. — Analyses are as described for Supplementary file 3, but are applied to principal components of the electrophysiological features of SCs. [file elife-52258-supp15.docx]

|  | | | **Fixed effects** | | | | **raw p** | | | **adjusted p** | | |
| --- | --- | --- | --- | --- | --- | --- | --- | --- | --- | --- | --- | --- |
| **property** | **N** | **n** | **Int** | **dvloc** | **housing** | **dv:housing** | **dvloc** | **housing** | **dv:housing** | **dvloc_adj** | **housing_adj** | **dv:housing_adj** |
| PC1 | 25 | 487 | 2.055 | -2.373 | 0.9582 | -0.0755 | 2.7e-86 | 0.0098 | 0.77146 | 3.2e-85 | 0.039 | 0.8890 |
| PC2 | 25 | 487 | -1.859 | 1.067 | 1.2929 | -0.1590 | 2.1e-07 | 0.0045 | 0.68647 | 1.2e-06 | 0.027 | 0.8890 |
| PC3 | 25 | 487 | -0.157 | 0.301 | -0.2278 | -0.0681 | 6.3e-02 | 0.4571 | 0.81496 | 1.5e-01 | 0.609 | 0.8890 |
| PC4 | 25 | 487 | 0.754 | -0.369 | -0.6950 | 0.1501 | 1.1e-01 | 0.1441 | 0.67227 | 2.2e-01 | 0.247 | 0.8890 |
| PC5 | 25 | 487 | 0.284 | -0.114 | -0.1393 | -0.0016 | 2.3e-01 | 0.6192 | 0.99355 | 4.0e-01 | 0.681 | 0.9935 |
| PC6 | 25 | 487 | 0.436 | -0.238 | -0.4509 | 0.3286 | 8.8e-01 | 0.2440 | 0.21869 | 9.4e-01 | 0.366 | 0.6561 |
| PC7 | 25 | 487 | 0.398 | -0.279 | -0.4526 | 0.3226 | 3.1e-01 | 0.0991 | 0.02446 | 4.6e-01 | 0.238 | 0.0978 |
| PC8 | 25 | 487 | 0.277 | -0.057 | -0.3795 | 0.0706 | 8.7e-01 | 0.0037 | 0.59357 | 9.4e-01 | 0.027 | 0.8890 |
| PC9 | 25 | 487 | 0.134 | -0.177 | -0.0042 | 0.0657 | 1.3e-02 | 0.6239 | 0.56108 | 3.8e-02 | 0.681 | 0.8890 |
| PC10 | 25 | 487 | 0.097 | -0.138 | -0.1354 | 0.2118 | 9.4e-01 | 0.0430 | 0.00576 | 9.4e-01 | 0.129 | 0.0345 |
| PC11 | 25 | 487 | 0.045 | -0.039 | -0.0537 | 0.0535 | 8.8e-01 | 0.8677 | 0.41403 | 9.4e-01 | 0.868 | 0.8890 |
| PC12 | 25 | 487 | -0.076 | 0.046 | 0.2241 | -0.1870 | 3.7e-03 | 0.1242 | 0.00056 | 1.5e-02 | 0.247 | 0.0067 |
