# Supplementary material for: Inter- and intra-animal variation in the integrative properties of stellate cells in the medial entorhinal cortex
Source: eLife. 2020 Feb 13;9:e52258. doi: 10.7554/eLife.52258 (PMC7067584; doi:10.7554/eLife.52258)
Supplement: Supplementary file 16. — Analyses are as for Supplementary file 10, but are applied to principal components of the electrophysiological features of SCs. [file elife-52258-supp16.docx]

| **component** | **deviance (mixed)** | **deviance (linear)** | **df (mixed)** | **df (linear)** | **p** | **p_adj** |
| --- | --- | --- | --- | --- | --- | --- |
| PC1 | 1463.85 | 1596.78 | 7 | 5 | 1.37e-29 | 3.28e-29 |
| PC2 | 1260.20 | 1658.40 | 7 | 5 | 3.40e-87 | 4.09e-86 |
| PC3 | 1394.81 | 1567.87 | 7 | 5 | 2.63e-38 | 1.05e-37 |
| PC4 | 1180.61 | 1441.66 | 7 | 5 | 2.07e-57 | 1.24e-56 |
| PC5 | 1217.87 | 1319.67 | 7 | 5 | 7.83e-23 | 1.57e-22 |
| PC6 | 1104.30 | 1237.28 | 7 | 5 | 1.33e-29 | 3.28e-29 |
| PC7 | 1070.53 | 1078.31 | 7 | 5 | 2.05e-02 | 2.46e-02 |
| PC8 | 904.79 | 929.29 | 7 | 5 | 4.78e-06 | 7.17e-06 |
| PC9 | 827.72 | 881.34 | 7 | 5 | 2.27e-12 | 3.89e-12 |
| PC10 | 590.98 | 592.69 | 7 | 5 | 4.25e-01 | 4.25e-01 |
| PC11 | 432.20 | 434.54 | 7 | 5 | 3.12e-01 | 3.40e-01 |
| PC12 | 116.24 | 128.36 | 7 | 5 | 2.34e-03 | 3.12e-03 |
